# Logical inconsistencies in time trade-off valuation of EQ-5D-5L health states: Whose fault is it?

**DOI:** 10.1371/journal.pone.0184883

**Published:** 2017-09-21

**Authors:** Zhihao Yang, Jan van Busschbach, Reinier Timman, M. F. Janssen, Nan Luo

**Affiliations:** 1 Section of Medical Psychology and Psychotherapy, Erasmus University Rotterdam, Rotterdam, the Netherlands; 2 Health Services Management Department, Guizhou Medical University, Guiyang, China; 3 The EuroQol Group, Rotterdam, the Netherlands; 4 Saw Swee Hock School of Public Health, National University of Singapore, Singapore; La Trobe University, AUSTRALIA

## Abstract

**Introduction:**

Inconsistency in the time trade-off (TTO) task in EQ-5D-5L occurs when a respondent gives a higher value to a logically worse health state, the occurrence of inconsistency compromises the quality of the data. It is not yet clear which factors are associated with individual level inconsistency. Relating inconsistency to the characteristics of the respondent, interviewer, and the interview process could be helpful in understanding the causes of inconsistency. The objective of this paper is to discover the factors associated with individual level inconsistencies.

**Methods:**

Twenty interviewers interviewed 1,296 respondents and each respondent valued 10 health states using the EQ-VT platform in 5 cities in China. At the respondent level, inconsistency was identified in terms of severity and quantity and related to the respondent’s background characteristics, the time and iterations spent on the wheelchair example task, and the formal TTO tasks, using multilevel multinomial regression analyses. Interviewers’ impact on inconsistencies was analyzed using single level multinomial regression analyses.

**Results:**

In the full dataset, slight inconsistency was more related to the interview process (Time spent on TTO task: RRR = 1.246 with 95%CI: 1.076,1.441; time spent on Wheelchair example: RRR = 0.815 with 95%CI:0.699,0.952) while severe inconsistency was more related to respondent’s gender (Gender: RRR = 2.347 with 95%CI:1.429,3.855). One Interviewer (Interviewer 7: RRR = 7.335 with 95%CI:1.908,28.195) and interviewer’s experience (Sequence: RRR = 0.511 with 95%CI:0.385,0.678) in general showed strong influence over inconsistency in the TTO task.

**Conclusion:**

In conclusion, logical inconsistency in the valuation of EQ-5D-5L health states is associated not only with respondents’ characteristics but also with interviewers’ performance and the interview process. The role of interviewers and the importance of interviewer training may be more crucial than hitherto believed. This finding could be generalizable to other interviewer-administered health-state valuation study.

## Introduction

EQ-5D-5L is a preference-based quality of life instrument which is mainly designed to generate health-state utility values that are required for calculation of quality-adjusted life years (QALYs) and cost-utility analysis [1]. With a classification system consisting of five dimensions (mobility, self-care, usual activities, pain/discomfort and anxiety/depression) and five levels of severity for each dimension (1 = no problems, 2 = slight problems, 3 = moderate problems, 4 = severe problems and 5 = extreme problems), the instrument defines (5^5^) = 3,125 unique health states, each of which can be represented using a 5-digit number or vector between 11111 (no problems in any dimension) and 55555 (extreme problems in all five dimensions). An important component of the instrument is the social tariff or value set that contains the utility values for all the health states it defines. With the value set available investigators can easily obtain the utility values of the EQ-5D-5L health states of interest, or find the utility values for their study populations by describing their health using the EQ-5D-5L classification system.

Establishing the value set for a preference-based health related quality of life instrument is not a trivial task. The general approach is to elicit the utility values for a subset of the health states defined by the instrument and develop a regression model to predict the values for all the health states, including those not directly valued. In the case of EQ-5D-5L, the currently recommended study protocol [2] requires 1,000 or more members of the general public each to value 10 different health states using the time trade-off (TTO) technique. After the TTO task, the current EuroQol Valuation Technology (EQ-VT) protocol also includes 7 pairs of discrete choice experiment (DCE) for each respondent. A number of countries have used the study protocol to establish their local EQ-5D-5L value sets [3,4]. In this paper, we focus mainly on the TTO task.

One issue that has occurred in the valuation of EQ-5D-5L health states is that some respondents give logically inconsistent values. That is, better health states are valued as more undesirable than worse health states [5]. For example, the state 11121 is valued lower than the state 22321. Logical inconsistency could be due to random mistake, however, if it occurs among a large proportion of respondents, it could signify the failure in the way the valuation technique is implemented. Regardless of the reason, such data lowers the precision of the estimated values. Specifically, logical inconsistency may attenuate the differences in values between health states [6] and consequently lead to underestimated health improvements when the values are used in cost-utility analysis [7]. In some valuation studies, inconsistent observations were excluded when constructing the value set, thereby potentially affecting representativeness if certain sub-groups of respondents score more inconsistencies than others [5,7–9]. Hence the magnitude of this issue and the underlying reasons should be investigated and, if possible, interventions should be implemented to minimize the potential bias caused by inconsistency.

Previous EQ-5D-3L valuation studies found that older and less-educated respondents were more likely to make inconsistent valuations [6,9]. EQ-5D-3L is similar to EQ-5D-5L except that there are only three descriptive levels for each dimension (no problems, moderate problems, and extreme problems). This result is not surprising as logical inconsistency could be due to poor understanding or misinterpretation of the valuation task [10,11]. However, it is not clear whether this is the case in the valuation of EQ-5D-5L health states and to what extent logical inconsistency is related to interviewers. In EQ-5D-5L valuation studies, interviewers play an important role in the conduct of the valuation tasks, and they are trained to follow a standardized protocol. Nevertheless, interviewer effects have been observed in previous studies [12].

The aim of the present study was to ascertain the factors underlying individual-level logical consistency in an EQ-5D-5L valuation study. We hypothesized that logical inconsistency was related to multiple factors with respect to interviewers, the interview process, and respondents’ background characteristics.

## Methods

### Data source

This study makes use of data collected in the EQ-5D-5L valuation study in China. The purpose of the valuation study was to establish the EQ-5D-5L value set in China from a societal perspective. The target population was urban residents in China [13]. Detailed description of the valuation study have been published elsewhere [13]. In the valuation study, the EQ-5D-5L was translated through a response scaling approach, which ensured the Chinese descriptors have similar interpretations with English counterpart [14]. Briefly, the study recruited members of the general population from five cities, namely: Beijing, Nanjing, Shenyang, Chengdu, and Guiyang [13]. In each city, members of the general population were recruited from a number of public places including community centers, parks, shopping centers, and university campuses. Sampling quotas were applied so that the sample resembled the target population in terms of age, sex, and education [13,15]. Inform consent was given to the respondent before conducting the interview [13], and ethics approval was not needed for this study in China as the valuation task is not seen as a medical intervention. Each respondent was interviewed face-to-face by a trained interviewer using the EQ-VT platform [16]. The interview had four sections. The first section was for respondents to report their own health using the EQ-5D-5L questionnaire, and their experience with serious illness. The second section asked respondents to complete 10 TTO tasks, each valuing a different EQ-5D-5L health state. The third section contained a set of discrete choice questions designed for valuation of selected EQ-5D-5L health states based on random utility theory. Data collected in this section was not used in the present study. The fourth section assessed respondents’ socio-economic and other background characteristics.

The ‘composite’ TTO technique was used in the study. This employs conventional TTO and lead-time TTO [17] to value better-than-dead and worse-than-dead states, respectively. The two TTO variants are described in detail elsewhere [18]. Briefly, conventional TTO elicits the raw value x (0 ≤ x ≤ 10) at which the respondent is indifferent between two alternatives: 1) living in full health for x years, and 2) living in an EQ-5D-5L health state for 10 years. The utility value is given by x/10. For health states considered to be worse than dead, the two alternatives in the valuation task are: 1) living in full health for x years, and 2) living in full health for 10 years and then in an EQ-5D-5L health state for another 10 years. The utility value is given by x/10–1.

At the interviews, the interviewer demonstrated and explained how the composite TTO works to the respondent using the state of ‘in a wheelchair’ as an example, before proceeding to the formal TTO tasks for the valuation of 10 different EQ-5D-5L health states [2]. The EQ-VT platform was designed to value a total of 86 EQ-5D-5L health states considered sufficient for the estimation of a value set. These 86 health states were divided into 10 blocks in such a way that each block consisted of the worst state (55555), one of the five mildest states (21111, 12111, 11211, 11121, 11112), and eight other unique health states. Each respondent was randomized to value one block of health states which were presented to the respondent in a random order.

A total of 20 interviewers, 4 for each city, conducted the interviews [13]. The interviewers were students and researchers from local universities. They were trained at a full-day workshop by their respective site project leaders who were trained in the same way by the principal investigator. The training focused on the use of a standardized protocol to conduct the interview, the principles of the TTO technique, and the objectives of the valuation study. As the TTO task was difficult to conduct, interviewers were instructed to perform multiple ‘practice’ interviews during and after the workshop with their peers and friends or family members.

### Measures of inconsistency

At the respondent level, the magnitude of logical inconsistency was assessed using three indicators: inconsistency rate, distance, and ΔTTO. Inconsistency rate was the number of inconsistently valued pairs of health states divided by all possible logical pairs. Inconsistency distance was calculated as the sum of the squared difference in levels for corresponding dimensions of the two health states involved. For example, the level differences between health states 12344 and 44444 were respectively 3, 2, 1 in the first three dimensions and 0 in the latter two, and thus the distance was 3^2^ + 2^2^ + 1 = 14. ΔTTO was the difference in utility values of two inconsistently valued health states. For example, if one respondent gave 21222 a utility 0.8 and 11112 a utility 0.5, the ΔTTO of this inconsistency would be 0.3.

Owing to the highly skewed distribution of inconsistency in all 3 indicators across respondents, as in other studies [6,10], respondents were categorized into 3 levels: none, slight, and severe. ‘None’ was defined as no observed inconsistency; ‘severe’ was defined as inconsistency rate higher than 10%, average inconsistency ΔTTO larger than 0.2, and average inconsistency distance larger than 9; and ‘slight’ was applied for respondents whose inconsistency profiles were neither ‘none’ nor ‘severe’[8,19]. So, a respondent is classified as severe inconsistent if he/she made more inconsistencies and those inconsistencies were more severe.

### Data analysis

Inconsistency factors studied included respondents’ demographic characteristics, interviewer identity, and interview process indicators. Respondents’ characteristics were age (16–24 years, 25–34 years, 35–44 years, 45–54 years, 55–64 years, 65-74years, ≥75 years), gender, and education (primary or lower, junior high school, senior high school, college or university, Masters or PhD). Interview process indicators were: time spent on the wheelchair example, number of iterations in the wheelchair example, and time spent on the 10 TTO tasks. The number of iterations indicated how many steps a respondent had moved before the indifferent point was reached in a TTO task. The number of iterations and the time spent on the wheelchair example, and the formal TTO tasks may reflect to what extent respondents and interviewers were engaged in the valuation tasks.

An additional process characteristic examined was the sequence of the interviews, that is, the rank order of the interviews conducted by the same interviewer in terms of the interview date and time. It was hypothesized that there was a learning curve for the interviewers in the study such that the quality of the interviews increased with the number of interviews that an interviewer completed. As a result, more interview experience would lead to a lower level of logical inconsistency.

A two-level multi-nominal logistic model (Eq 1) with the interviewer as the upper level and the respondent as the lower level was used to explore logical inconsistency factors. This model estimated the average effects of the lower-level factors among the interviewers. The requirement to discern levels was determined using likelihood ratio tests [20]. Age, gender, education level (edu), interview sequence, TTO time, TTO iteration (ttoit), wheelchair time and wheelchair iteration were entered as covariates. The covariates sequence, times, and iterations were standardized (by dividing the raw data with its Standard Error) in order to enhance interpretation of the relative risk ratios (RRR) for category i compared to the reference category no inconsistencies. A RRR > 1 suggests an increased risk of that outcome compared to the reference group. A RRR between 0 and 1 suggests a reduced risk compared to the reference group.

$$RRR=e^{\beta_{00}+u_{0j}+\beta_{1i}age+\beta_{2i}edu+\cdots +\beta_{8i}ttoit}$$ (1)

Where β_00_ is the overall mean intercept and u_0j_ is the random intercept to identify clusters, here: interviewers.

Additional analysis determined whether there were differences in inconsistencies between the interviewers. As ‘interviewer’ was included as a between-subject factor in this analysis, a single-level multi-nominal regression model (Eq 2) which included both interviewer and the above-mentioned covariates was used. Relative risk ratios, their 95% confidence intervals, and p-values of the independent variables were estimated using STATA version 13.1. Covariates were deleted in a backward procedure, with p>0.05 as the criterion for deletion. Interaction terms between statistically significant covariates were created and examined based on the results of the two models.

$$RRR=e^{\beta_{0i}+\beta_{1i}age+\beta_{2i}edu+\cdots +\beta_{8i}ttoit+\beta_{9i}inter2+\cdots +\beta_{23i}inter20}$$ (2)

## Results

### Data description

Of 1,302 participants in the valuation study, 1,296 finished the interview. Each of the 20 interviewers conducted at least 50 interviews. Table 1 summarizes the demographic information of the interviewees and the summarized information of the interview process.

**Table 1:** Demographic information of interviewees and the summarized information of interview process.

	Total sample
**Age group (years)**	(N, %)
16–24	235, 18%
25–34	231, 18%
35–44	237, 18%
45–54	258, 20%
55–64	222, 17%
65–74	79, 6%
≥75	34, 3%
**Gender**	(N, %)
Male	650, 50%
Female	646, 50%
**Education**	(N, %)
Primary or Lower	138, 11%
Junior high school	405, 31%
Senior high school	462, 36%
College or University	225, 17%
Masters or PhD	66, 5%
**Interview Sequence (Rank orders)**	(Mean, SD)
	33.4,19.6
**Time spent on TTO task (Minutes)**	(Mean, SD)
	14.2,5.3
**Time spent on Wheelchair example task (Minutes)**	(Mean, SD)
	6.3,3.2
**Iterations spent on TTO task (steps)**	(Mean, SD)
	7.9,2.5
**Iterations spent on Wheelchair example task (steps)**	(Mean, SD)
	22.1,11.9

Out of 1,296 respondents, 723 (56%) did not display any inconsistency; the remaining 44% gave at least one inconsistent response. The numbers of respondents who were ‘slightly’ and ‘severely’ inconsistent amounted to 499 and 74 respectively. The rate, distance, and ΔTTO of logical inconsistency are summarized in Table 2.

**Table 2:** Inconsistency severity measured by three criteria.

Measurement criteria	Severity degree	Numbers identified	Total inconsistency rate	Average inconsistency distances	Average inconsistency ΔTTO
Inconsistency rate	Slight	447	0.045	14.287	0.235
	Severe	126	0.169	22.713	0.333
Inconsistency distance	Slight	160	0.040	4.966	0.254
	Severe	413	0.085	20.469	0.257
Inconsistency ΔTTO	Slight	325	0.059	15.317	0.096
	Severe	248	0.090	17.219	0.467
Inconsistency fulfilled all criteria	Slight	499	0.056	14.946	0.223
	Severe	74	0.189	24.194	0.482

### Factors associated with inconsistency

Significant variables associated with logical inconsistency and their effects in the two-level model are displayed in Table 3. The likelihood ratio test showed that both levels (interviewers and respondents) were statistically significant (P <0.01). Three variables were significantly associated with slight inconsistency and another two variables were associated with severe inconsistency (Table 3). Specifically, more time spent on the wheelchair example, less time spent on the TTO task, and interviews completed at a later sequence, were associated with less likelihood of slight inconsistency; female respondents, and interviews completed at a later sequence were associated with less likelihood of severe inconsistency. The RRR is interpreted as, for example, compared to reference group, the risk of being slightly inconsistent is 1.246 times higher for every one unit of more time spent on TTO task.

**Table 3:** Inconsistency: Multi-level multinomial logistic model in full dataset, N = 1,296.

Variables	RRR(unadjusted)	95%CI	RRR (adjusted)	95% CI
**0 (Reference level: no inconsistency)**	Base outcome		Base outcome	
**1 (Slight inconsistency)**				
Sequences (Rank orders)	0.810**	0.720, 0.912	0.806**	0.707, 0.918
Standardized time spent on TTO task	1.081	0.957, 1.220	1.246**	1.076, 1.441
Standardized time spent on wheelchair example	0.855*	0.755, 0.967	0.815*	0.699, 0.952
**2 (Severe inconsistency)**				
Sex	1.997**	1.230, 3.243	2.347**	1.429, 3.855
Sequences (Rank orders)	0.540**	0.417, 0.699	0.511**	0.385, 0.678

“Sex” is coded “0” for female respondent, and “1” for male respondent.

** Significant at 0.01 level.

* significant at 0.05 level.

Two interviewers were found to be associated with a higher likelihood of slight and/or severe logical inconsistency in the single-level model (Table 4). One of the interviewers was particularly unusual as the relative risk ratio were found to be much higher compared to those conducted by an averagely performed interviewer, after adjusting for covariates. Interaction terms (i.e. education level of respondent*interviewer) were explored and proved less interesting in terms of statistical significance.

**Table 4:** Interviewer effect on inconsistency: Multinomial logistic model in full dataset, N = 1,296.

Variables	RRR (unadjusted)	95% CI	RRR (adjusted)	95% CI
**0 (Reference level: no inconsistency)**	Base outcome		Base outcome	
**1 (Slight inconsistency)**				
Interviewer 7	3.486**	1.506, 8.071	3.476**	1.475, 8.191
Interviewer 9	2.242*	1.073, 4.683	2.659*	1.241, 5.696
**2 (Severe inconsistency)**				
Interviewer 7	8.054**	2.205, 29.411	7.335**	1.908, 28.195

Dummy variables ‘interviewer’ represent different interviewers, the reference level is ‘interviewer1’ from Shenyang, whose inconsistency level is the median among all interviewers.

** Significant at 0.01 level.

* significant at 0.05 level.

## Discussion

As hypothesized, the factors interviewer, interview process, and respondent were all related to individual level logical inconsistency in the valuation of EQ-5D-5L health states. In terms of respondents’ characteristics, male gender was associated with severe logical inconsistency. One explanation could be that male respondents might have had poorer engagement than females in the present study. In the previous EQ-5D-3L valuation study conducted in China, young and well-educated respondents were more likely to give inconsistent TTO answers [21]. Unlike previous studies [6,9,10], older age was not associated with logical inconsistency in the present valuation study. This could be due to the efficiency of the survey tool: a computerized software program was used to demonstrate the valuation tasks in the EQ-5D-5L valuation study while a time board was used in previous studies. It should be noted that respondents’ characteristics such as gender are not modifiable factors in valuation studies aiming at establishing a societal value set. For such studies, samples should be representative of the general population in terms of demographics. Hence, respondents who are more susceptible to logical inconsistency, cannot be removed from EQ-5D-5L valuation studies; the only intervention is to have interviewers pay more attention to these respondents.

More importantly, we found that interviewer and interview process indicators were independently associated with logical inconsistency. Specifically, interviews conducted by certain interviewers, those conducted earlier on by interviewers (sequence effect), and those in which less time was spent on the wheelchair example, suffered more from this issue. The variations across interviewers suggest that some interviewers did not perform to the expected standards. This could be due to poor understanding of the valuation tasks or poor compliance to the interview protocol. The sequence effect suggests that interviewers might still have been on a learning curve, that is, they had not been versed enough in conducting the interviews at the time they started. Wheelchair time might be an indicator of training adequacy: when this was inadequate, logical inconsistency would increase. It is notable that the more time spent on TTO tasks, the more inconsistency occurred. One explanation could be that if the respondents did not understand or engage in the task, it took them longer to finish the TTO tasks while this did not warrant consistent responses.

Therefore, our study supports the extension of EQ-5D-5L valuation protocol with a quality control (QC) tool [22]. It also should be noted that this data collection was done in the first version of EQ-VT protocol. The new protocol with the several modification to the original protocol, including the QC process lower the inconsistency rate from 11% to 3% [22]. By using the new valuation protocol with QC tool, individual interviewers are monitored during the entire data collection period for their performance including time spent on explaining the wheelchair example [22]. This monitoring is possible because the information is collected by the survey program and uploaded by interviewers on a daily basis. Nevertheless, our study suggests that future EQ-5D-5L valuation studies could benefit from more training for interviewers. In addition, our findings could be generalizable to other interviewer-administered health-state valuation study. The role of interviewers and the importance of interviewer training might be more crucial than hitherto considered, especially for the valuation study that is done without proper QC process during the data collection.

This study raised the question concerning how to handle logical inconsistency in establishing an EQ-5D-5L value set: should the inconsistent data be removed? Past studies showed that keeping inconsistent data will attenuate the differences in values between health states [23]. On the other hand, if inconsistent responses are systematically higher in certain groups of respondents (e.g. male respondents), removing these data will affect the representativeness of population samples [5]. Only a few EQ-5D-3L value sets were estimated by excluding some of the logically inconsistent data [7,19,21]. Nevertheless, it can be postulated that values of extreme health states may be biased if logical inconsistency occurs with respect to these states. For example, good health states are unlikely to be overestimated because the logical inconsistency is one-sided: such health states are more likely to be valued lower rather than higher because the valuation tasks are designed in a way that no health states can be valued as > 1.0, the upper bound of utility value. Hence it is advisable to assess the effect of logical inconsistency on the estimated EQ-5D-5L value set.

One limitation of this study is that we limited our analysis of logical inconsistency to logistic analysis due to the skewed distributions of inconsistency at individual level. Moreover, the classification of inconsistency in the logistic model was arbitrary. There is no a well-accepted definition for ‘slight’ inconsistency or ‘severe’ inconsistency. However, in this study, in order to identify between “those who made careless mistakes” and “those who seem do not understand the task at all”, the line was drawn.

In conclusion, logical inconsistency in the valuation of EQ-5D-5L health states is associated not only with respondents’ characteristics but also with interviewers’ performance and the interview process. Our study has highlighted the importance of interviewers for health-state valuation using the TTO elicitation procedure.

## Supporting information

S1 File Individual level inconsistency. (DTA)
